# Two Type VI Secretion Systems of *Enterobacter cloacae* Are Required for Bacterial Competition, Cell Adherence, and Intestinal Colonization

**DOI:** 10.3389/fmicb.2020.560488

**Published:** 2020-09-24

**Authors:** Jorge Soria-Bustos, Miguel A. Ares, Carlos A. Gómez-Aldapa, Jorge A. González-y-Merchand, Jorge A. Girón, Miguel A. De la Cruz

**Affiliations:** ^1^Unidad de Investigación Médica en Enfermedades Infecciosas y Parasitarias, Hospital de Pediatría, Centro Médico Nacional Siglo XXI, Instituto Mexicano del Seguro Social, Mexico City, Mexico; ^2^Departamento de Microbiología, Escuela Nacional de Ciencias Biológicas, Instituto Politécnico Nacional, Mexico City, Mexico; ^3^Instituto de Ciencias Básicas e Ingeniería, Universidad Autónoma del Estado de Hidalgo, Carretera Pachuca-Tulancingo Km 4.5 Mineral de la Reforma, Hidalgo, Mexico; ^4^Centro de Detección Biomolecular, Benemérita Universidad Autónoma de Puebla, Puebla, Mexico

**Keywords:** *E. cloacae*, T6SS, ClpV, Hcp, virulence

## Abstract

*Enterobacter cloacae* has emerged as an opportunistic pathogen in healthcare-associated infections. Analysis of the genomic sequences of several *E. cloacae* strains revealed the presence of genes that code for expression of at least one type VI secretion system (T6SS). Here, we report that *E. cloacae* strain ATCC 13047 codes for two functional T6SS named T6SS-1 and T6SS-2. T6SS-1 and T6SS-2 were preferentially expressed in tryptic soy broth and tissue culture medium (DMEM), respectively. Mutants in T6SS-1-associated genes *clpV1* and *hcp1* significantly affected their ability of inter- and intra-bacterial killing indicating that T6SS-1 is required for bacterial competition. In addition, the Hcp effector protein was detected in supernatants of *E. cloacae* cultures and a functional T6SS-1 was required for the secretion of this protein. A *clpV2* mutant was impaired in both biofilm formation and adherence to epithelial cells, supporting the notion that these phenotypes are T6SS-2 dependent. *In vivo* data strongly suggest that both T6SSs are required for intestinal colonization because single and double mutants in *clpV1* and *clpV2* genes were defective in gut colonization in mice. We conclude that the two T6SSs are involved in the pathogenesis scheme of *E. cloacae* with specialized functions in the interaction with other bacteria and with host cells.

## Introduction

*Enterobacter cloacae* are Gram-negative, facultative anaerobic and rod-shaped bacteria belonging to the order *Enterobacterales* (McAdam, 2020). These bacteria are saprophytic in the environment and are also part of the human gut microbiota (Mezzatesta et al., 2012). Currently, six species of the *Enterobacter cloacae* complex have been described: *E. asbuariae*, *E. cloacae*, *E. hormaechei*, *E. kobei*, *E. ludwigii*, and *E. nimipressuralis* (Paauw et al., 2008). *E. cloacae* is a human opportunistic pathogen that is frequently associated with hospital-acquired infections of the lower respiratory tract, urinary tract, and meninges (Liu et al., 2013). Despite the relevance of *E. cloacae* as a nosocomial pathogen, its pathogenicity mechanisms are not yet fully understood, but biofilm formation is a virulence feature of this opportunistic microorganism (Zhou et al., 2014; Zurob et al., 2019). In addition, some *E. cloacae* strains possess cytotoxic activity, suggesting the secretion of bacterial toxins to the cell host (Barnes et al., 1997; Krzyminska et al., 2009).

Successful host colonization by a bacterial pathogen depends on functional secretory systems, which translocate and secrete several effectors proteins into host cells or to the extracellular environment, respectively, with the purpose of competing with the host microbiota (Pukatzki et al., 2006). The type 6 secretion system (T6SS) is a multi-proteinaceous complex encoded by large gene regions found in 25% of all sequenced Gram-negative bacteria (Boyer et al., 2009). Interestingly, the T6SS core proteins share structural homology with proteins that form the T4 bacteriophage tail (Bingle et al., 2008; Boyer et al., 2009; Ho et al., 2014).

At least 13 core components (*tssA* to *tssM*) are required for the assembly of a functional T6SS (Cherrak et al., 2019; Navarro-Garcia et al., 2019). The tail-like structure is formed of Hcp (hemolysin-coregulated protein) homo-hexamers, which are heaped and enveloped by heterodimers of TssB/TssC proteins forming a sheath-type structure (Mougous et al., 2006; Zoued et al., 2014). Located at the tip of the inner tube formed by the Hcp homo-hexamers, the VgrG/PAAR complex functions as a cell-puncturing device for injection of multiple effectors into target cells (Shneider et al., 2013; Douzi et al., 2016). The ClpV protein is an AAA^+^ ATPase that disassembles the contracted tubule, recycling different components of the tail-like structure, maintaining the membrane complex stability, which can be reused for multiple sheath-like assemblies (Kube et al., 2014).

**FIGURE 1:**
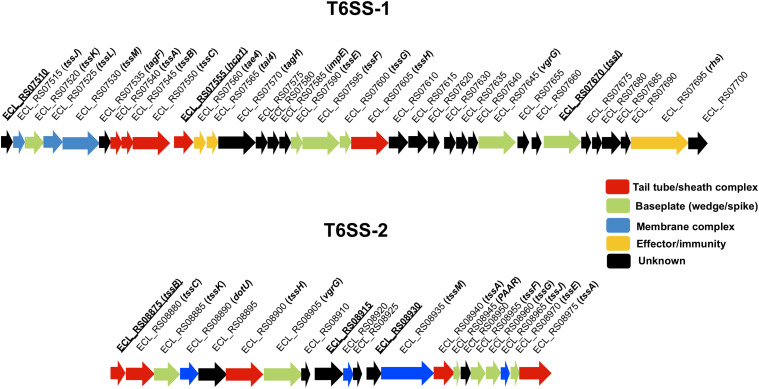
Genetic organization of T6SS-1 and T6SS-2 of *E. cloacae* ATCC 13047. Genes are plotted as arrows in order according to their genomic positions. The underline text represents the genes whose expression was quantified by RT-qPCR and are the first genes of putative polycistronic operons. Tail tube/sheath complex, baseplate, membrane complex, effector/immunity, and unknown functions are colored in red, green, blue, yellow, and black, respectively.

Different studies have described the role of T6SS in virulence, immunomodulation, persistence, adherence, and invasion to epithelial cells, biofilm formation, and inter-bacterial competition (Ma et al., 2009; Hood et al., 2010; de Pace et al., 2011; Lertpiriyapong et al., 2012; Weyrich et al., 2012; Russell et al., 2014; Repizo et al., 2015; Gallique et al., 2017). The role of T6SS in *E. cloacae* remains unknown and so far has not been implicated in the virulence of this opportunistic pathogen. Recent comparative genome analysis of different *E. cloacae* strains showed that *E. cloacae* ATCC 13047 possesses two different T6SS-like clusters (Liu et al., 2013) that we named T6SS-1 and T6SS-2 (Figure 1), suggesting that the presence of two T6SS could provide adaptive advantages for *E. cloacae* in bacterial competition and virulence, as it has been reported for other bacteria that possess two or more T6SS clusters (Journet and Cascales, 2016; Chen et al., 2019).

In this work, we described the role of both *E. cloacae* T6SS-1 and T6SS-2 in inter-bacterial competition, biofilm formation, adherence to cells, and intestinal colonization. We found that T6SS-1 is required for inter-bacterial competition because mutants of *E. cloacae* deficient in T6SS-1 were not able to kill *E. coli* and another Gram-negative enterobacteria as the parental strain did. Virulence phenotypes such as biofilm formation and cell adherence were found to be T6SS-2 dependent. Finally, *in vivo* studies showed that both T6SSs are required for the colonization of mice gut. Our results show that both T6SSs are virulence factors that confer *E. cloacae* the ability to survive in different environments and ecological niches and colonize different hosts.

## Materials and Methods

### Bacterial Strains and Culture Conditions

Bacterial strains and plasmids used in this study are listed in Table 1. To analyze the expression of T6SS genes, several bacteriological broths such as lysogenic broth (LB), trypticase soy broth (TSB), Dulbecco’s Modified Eagle’s Medium (DMEM) with high glucose (4.5 g/l), pleuropneumoniae-like organisms (PPLO) broth, and colonization factor antigen (CFA) broth were used. When necessary, media were supplemented with antibiotics: ampicillin (200 μg/ml), kanamycin (50 μg/ml), and tetracycline (10 μg/ml).

**TABLE 1:** Bacterial strains and plasmids used in this study.

**Strain or plasmid**	**Description**	**References**
**Strains**		
*E. cloacae* WT	Wild-type *E. cloacae* strain ATCC 13047	ATCC
*E. cloacae* Δ*clpV1*	*E. cloacae* Δ*clpV1*:FRT	This study
*E. cloacae* Δ*clpV2*	*E. cloacae* Δ*clpV2*:FRT	This study
*E. cloacae* Δ*clpV1* Δ*clpV2*	*E. cloacae* Δ*clpV1*:FRT *clpV2*:FRT	This study
*E. cloacae* Δ*hcp1*	*E. cloacae* Δ*hcp1*:FRT	This study
DH5α	*E. coli* K-12 laboratory strain	Invitrogen
EAEC	Enteroaggregative *E. coli* strain 042	Nataro et al., 1985
EPEC	Enteropathogenic *E. coli* strain E2348/69	Levine et al., 1978
ETEC	Enterotoxigenic *E. coli* strain E9034A	Levine et al., 1984
EHEC	Enterohemorrhagic *E. coli* strain EDL933	Riley et al., 1983
UPEC	Uropathogenic *E. coli* strain CFT073	Mobley et al., 1990
APEC	Avian-pathogenic *E. coli* strain MT78	Dho and Lafont, 1982
KPN	*K. pneumoniae* strain 123/01	Ares et al., 2016
KOX	*K. oxytoca* strain 1	Clinical isolate
STY	*S. enterica* serotype Typhi strain ATCC 6539	ATCC
STM	*S. enterica* serotype Typhimurium strain 12023	Laboratory collection
ECL11E	*E. cloacae* strain 11E	Clinical isolate
ECL18E	*E. cloacae* strain 18E	Clinical isolate
ECL31E	*E. cloacae* strain 31E	Clinical isolate
ECL43E	*E. cloacae* strain 43E	Clinical isolate
**Plasmids**		
pMPM-T3	p15A derivative low-copy number cloning vector, lac promoter Tc^*R*^	Mayer, 1995
pT3-ClpV1	pMPMT3 derivative expressing ClpV1 from the lac promoter	This study
pT3-ClpV2	pMPMT3 derivative expressing ClpV2 from the lac promoter	This study
pT3-Hcp1	pMPMT3 derivative expressing Hcp1 from the lac promoter	This study
pMPM-K6Ω	p15A derivative cloning vector, pBAD (ara) promoter; Km^*R*^	Mayer, 1995
pKD119	pINT-ts derivative containing the λ Red recombinase system under an arabinose-inducible promoter, Tc^*R*^	Datsenko and Wanner, 2000
pKD4	pANTsy derivative template plasmid containing the kanamycin cassette for λ Red recombination, Ap^*R*^	Datsenko and Wanner, 2000
pCP20	Plasmid that shows temperature-sensitive replication and thermal induction of FLP synthesis, Ap^*R*^, Cm^*R*^	Datsenko and Wanner, 2000

### Construction of *E. cloacae* Mutants

*Enterobacter cloacae* was targeted for mutagenesis of *clpV1*, *hcp1*, and *clpV2*, following the procedure previously reported (Datsenko and Wanner, 2000) with some modifications. Each purified PCR product was electroporated into competent *E. cloacae* carrying the lambda-Red recombinase helper plasmid pKD119, whose expression was induced by adding L-(+)-arabinose (Sigma) at a final concentration of 1.0%. PCR fragments containing *clpV1*, *clpV2*, and *hcp1* sequences flanking a kanamycin cassette were generated using gene-specific primer pairs (Table 2), and the pKD4 plasmid was used as template. The resistance genes were eliminated by using FLP recombinase from pCP20 plasmid. All mutations were verified by PCR and sequencing.

**TABLE 2:** Primers used in this study.

**Primer**	**Sequence (5′-3′)**	**Target gene**
**FOR qPCR**		
ECL_7510-5′	ACGCTTGTCACCGGTAAAAC	*ECL_RS07510*
ECL_7510-3′	TTGATTACCGCACGCATTGG	
ECL_7555-5′	TTGCTGTGGTGGATTTGTCG	*hcp1*
ECL_7555-3′	ACACCGGCTGGACTGATATTAC	
ECL_7670-5′	CGCATCGATTTCACGGTTATCC	*ECL_RS07670*
ECL_7670-3′	TTCACGCGGCCATATTTGTC	
ECL_8875-5′	AATGTGACGCTGCGCTTTTC	*ECL_RS08875*
ECL_8875-3′	AATTACGCATCGCCAGCATG	
ECL_8915-5′	TGGGGAGCGTGAAAAATGTG	*ECL_RS08915*
ECL_8915-3′	ATCATTGCCTGCGGTTTCAG	
ECL_8930-5′	TCCCGGGATTAACAGCCTTTC	*ECL_RS08930*
ECL_8930-3′	TTGCTGCTCCGTTTTCACTG	
rrsH-5′	CAGCCACACTGGAACTGAGA	*rrsH*
rrsH-3′	GTTAGCCGGTGCTTCTTCTG	
**For cloning**		
hcp1-*Hin*dIII-5′	ATC*AAGCTT*GAGTAGATATTATGGCTATTG	*hcp1*
hcp1-*Bam*HI-3′	CCC*GGATCC*ACCCACTACTATTATGCTTCTTTG	
clpV1-*Kpn*I-5′	GGG*GGTACC*TCCTTCCGTATACCGAATCATTGT	*clpV1*
clpV1-*Bam*HI-3′	GGG*GGATCC*GTCCGATTGTTATCATTATCCGTCA	
clpV2-*Kpn*I-5′	GGG*GGTACC*CGGGCGATGACCAGTCCAA	*clpV2*
clpV2-*Bam*HI-3′	GGG*GGATCC*CACCGAAAGCAGATGATGGC	
**For mutagenesis**		
Ecl-clpV1-H1P1	TCATTGTTAATGGAAACTAACATGTCAGAAATTAGCCGTGCCGTGTGTAGGCTGGAGCTGCTTCG	*clpV1*
Ecl-clpV1-H2P2	TTACAGTTCTCTTGATGATTATTACGCGGCAAACTGACAGTGAAACATATGAATATCCTCCTTAG	
Ecl-clpV2-H1P1	GGTGCTGTCGCTTAGGCGCTGGTGGACCGGCAGAGCCTGGTGCGCTGTAGGCTGGAGCTGCTTCG	*clpV2*
Ecl-clpV2-H2P2	ATCTCAGCGGGGGATTACTGCTCATGCGTGCGCTCCTTCTGTCGACATATGAATATCCTCCTTAG	
Ecl-hcp1-H1P1	CATCCACGAAGAGTAGATATTATGGCTATTGATATGTTTCTGAAGTGTAGGCTGGAGCTGCTTCG	*hcp1*
Ecl-hcp1-H2P2	GCCCTTTCGGACCCACTACTATTATGCTTCTTTGTTTTCTTTGATCATATGAATATCCTCCTTAG	

*Italic letters indicate the respective restriction enzyme site in the primer. The sequence corresponding to the template plasmid pKD4 is underlined.*

### Construction of Plasmids

Plasmids pT3-Hcp1, pT3-ClpV1, and pT3-ClpV2 were generated by cloning of a PCR product containing the corresponding *hcp1*, *clpV1*, and *clpV2* regions of *E. cloacae*, respectively, into the pMPM-T3 plasmid (see primers in Table 2). The PCR products were digested with *Hin*dIII/*Bam*HI for *hcp1*, and *Kpn*I/*Bam*HI for *clpV1* and *clpV2*. The digested PCR products were ligated into pMPM-T3, which was also previously digested with the same enzymes. The identity of the insert was confirmed by DNA sequencing.

### Quantitative RT-PCR

Total RNA extraction was performed using the hot phenol method as described (Jahn et al., 2008). DNA was removed with TURBO DNA-free (Ambion) and the quality of RNA was assessed using a NanoDrop (ND-1000; Thermo Scientific) and an Agilent 2100 bioanalyzer with a Picochip (Agilent Technologies). The absence of contaminating DNA was controlled by lack of amplification products after 35 qPCR cycles. cDNA was prepared using 1 μg of RNA, random hexamer primers (0.2 μg/μl), and a M-MulV-RT (20 U/μl, reverse transcriptase of Moloney Murine Leukemia Virus; Thermo Fisher Scientific). Specific primers were designed with the Primer3Plus software^1^ and are listed in Table 2. Quantitative RT-PCR was performed in a Lightcycler 480 instrument (Roche). Control reactions with no template (water) and minus-reverse transcriptase (RNA) were run with all reactions. 16S rRNA was used as a reference gene for normalization and the relative gene expression was calculated using the 2^−ΔΔ*Ct*^ method (Livak and Schmittgen, 2001). Expression of 16S rRNA remained unaffected in all conditions tested (Supplementary Figure S1). These experiments were performed in triplicate on three independent times.

### Bacterial Competition

Experiments were done essentially as previously described (Repizo et al., 2015), with some modifications. Briefly, the different *E. cloacae*, *E. coli*, *Klebsiella*, and *Salmonella* strains were grown overnight with aeration in 5 ml of LB broth containing the appropriate antibiotics, and overnight cultures were then adjusted up to OD_600 nm_ ∼1.0 and they were mixed in 4:1 ratio (predator/prey). Aliquots of 20 μl of the mixed bacterial culture were spotted onto LB agar and incubated at 37°C for 4 h. The bacterial spot on the agar surface was subsequently removed, vigorously resuspended in PBS, and the colony-forming units (CFU) per milliliter of surviving prey strains were measured by plating serial dilutions on solid selective media. The selective medium contained 50 μg/ml of kanamycin for prey strains previously transformed with pMPM-K6 plasmid. The output/input ratio of the prey to predator strains was interpreted as survival and includes a minimum of three independent assays.

### Hcp Secretion Analysis

*E. cloacae* strains were grown in TSB with aeration in 35 ml of LB broth at 37°C (containing the appropriate antibiotics) until OD_600 nm_ ∼1.0. Supernatants of culture (25 ml) were centrifuged at 4000 × *g* followed by filtration (0.22 μm) and concentration using Amicon Ultra-15 centrifuge filters. Concentrated supernatants were resuspended in Laemmli sample buffer (Bio-Rad) and analyzed by 16% SDS-PAGE. Polyacrylamide gels were stained using Coomassie Brilliant Blue.

### Liquid Chromatography–Tandem Mass Spectrometry (LC-MS/MS)

In-gel digestion and mass spectrometry (LC-MS/MS) were performed as previously described (Garcia-Morales et al., 2017). Protein bands were cut and treated with 10 mM DTT in 50 mM NH_4_HCO_3_ at 56°C for 45 min. After incubation, DTT was replaced by 55 mM iodoacetamide. The dried gel fragments were incubated at 50°C for 1 h with 50 mM NH_4_HCO_3_ containing 0.01% of ProteaseMAX Surfactant (Promega, United States). The resulting peptides were tested on an LC-MS system consisting of a fluid flow micro-chromatograph Accela with “spliter” (1/20) and a mass spectrometer LTQ-Orbitrap Velos (Thermo-Fisher, San Jose, CA, United States) with an electrospray ionization system. Fragmentation data were captured in a dependent manner according to predetermined loads with an isolation width of 3.0 (*m*/*z*), normalized collision energy of 35 arbitrary units, Q activation of 0.250, activation time of 40 ms, and a maximum injection time of 10 ms per micro-scan. The resultant MS/MS data were searched against the NCBI non-redundant database^2^, and the identification of proteins was determined by using Protein Prospector version 5.10.17 (San Francisco, CA, United States^3^). Mass tolerances for precursor ions and fragment ions were set to 20 ppm and 0.2 Da, respectively. In all cases, match punctuations were less than 5 ppm. A discriminant score was carried out for each analyzed peptide; this value is the combination of two measurements of the search result. One is the expectation value for the peptide match (“FDR,” a measure of the likelihood that a match is random) and the other is a “best peptide score,” which takes into account the fact that if a protein has been confidently identified in a sample, it is more likely that other peptides from the same protein will be identified (San Francisco, CA, United States see text footnote 3).

### Hemolysis Quantification

The quantification of bacterial hemolysis was performed as described (Soto et al., 2017). *E. cloacae* strains were grown in TSB until the OD_600 nm_ = 1.0, and 0.5 ml culture was added to 0.5 ml of a 4% (*v*/*v*) red blood cells (RBC)/saline solution (0.9% NaCl), and centrifuged at 2500 × *g* for 1 min. After 4 h of incubation at 37°C, the bacteria/RBC mix was resuspended, and cells were centrifuged at 12,000 × *g* for 1 min, and the hemoglobin released into the supernatant was determined by measuring the OD at 450 nm. The complete hemolysis was expressed in percentage with respect to the lysis obtained with wild-type *E. cloacae* strain. These experiments were performed in triplicate on three independent times.

### Biofilm Formation

Adhesion to abiotic surface (polystyrene) was analyzed using 96-well plates as described previously (Ares et al., 2016). Overnight cultures of bacteria grown in LB broth (10 μl) were added to 1 ml of DMEM. This volume was distributed in quintuples (100 μl per well) into a 96-well plate and incubated at room temperature for 24 h. To remove loosely attached bacteria, the culture was removed from the wells and gently rinsed three times with PBS and bound bacteria were stained with 1% crystal violet (CV) and incubated for 20 min at room temperature. After incubation, the wells were rinsed three times with phosphate buffered saline (PBS), and the dye was solubilized in 100 μl of ethanol 70%. Finally, the amount of extracted crystal violet was determined by measuring the OD_600_ using an ELISA Multiskan Plate reader (Thermo Scientific). These experiments were performed in triplicate on three independent times.

### Bacterial Adherence

Monolayers of HeLa (ATCC CCL-2) cell line (7 × 10^5^ cells/well) were infected with the indicated strains of an LB broth overnight culture at a multiplicity of infection (MOI) of 100. Epithelial cells were grown in DMEM with 10% fetal bovine serum (FBS). After infection, eukaryotic cells were incubated in DMEM with no FBS for 2 h at 37°C under an atmosphere of 5% CO_2_. After 2 h of incubation period, cells were washed three times with PBS and then lysed with a solution of 0.1% Triton X-100 for 15 min. After homogenization, the lysates containing total cell-associated bacteria were diluted serially in PBS and plated onto LB agar plates to enumerate adherent bacteria. The results shown are the mean of at least three experiments performed in triplicate on different days.

### Phagocytosis of Bacteria by Macrophages

THP-1 (ATCC TIB-202) human monocytes differentiated to macrophages with 200 nM of phorbol 12-myristate 13-acetate for 24 h (6 × 10^5^) were seeded into 24-well tissue culture plates. Bacteria were grown in 5 ml of LB broth overnight at 37°C. Macrophages were infected with a MOI of 100 in a final volume of 1 ml RPMI 1640 tissue culture medium supplemented with 10% heat-activated FBS. Plates were incubated at 37°C under a humidified 5% CO_2_ atmosphere. After 2 h, cells were rinsed three times with sterile PBS and incubated for an additional 60 min with 1 ml of RPMI 1640 containing 10% FBS and gentamycin (100 μg/ml) to eliminate extracellular bacteria. Cells were then rinsed three times with sterile PBS and lysed with 0.1% Triton X-100. After homogenization, serial dilutions were plated onto LB agar to enumerate total CFUs.

### Ethics Statement

Animal experimentation was conducted in strict accordance with good animal practice as defined by the use of laboratory animals and quality requirements, in agreement with animal welfare bodies from Mexico (SAGARPA NOM-062-ZOO-1999: “Technical Specifications for the Production, Care and Use of Laboratory Animals”). All animal work was approved by the Internal Ethics Committee of the Animal Resource Facility of the Universidad Autónoma del Estado de Hidalgo (approval number: CICUAL/016/2019R to Carlos A. Gómez-Aldapa).

### Mouse Inoculation Experiments

Mice infection experiments were performed using BALB/c strain. Mice groups (*n* = 6) were pretreated with 50 mg of streptomycin 24 h before infection with *E. cloacae* strains. Mice were infected by intragastric (i.g.) inoculation with 1 × 10^9^ CFU/ml of bacteria under sterile conditions. Fresh fecal pellets were collected directly into microtubes at 3 and 6 days post-infection (p.i.). Pellets were resuspended vigorously in sterile PBS 1×, and CFUs per gram of feces were determined by plating serial dilutions on LB agar plates with ampicillin (200 μg/ml).

### Statistical Analysis

Prism 5 (GraphPad) was used for statistical differences. One-way ANOVA followed by Tukey’s multiple comparison test and unpaired Student’s *t* test was performed. A *p-*value ≤ 0.05 was considered statistically significant.

## Results

### Identification and Expression Analysis of the T6SSs in *E. cloacae*

The genome of *E. cloacae* ATCC 13047 codes for two putative T6SS loci (Liu et al., 2013), which we named T6SS-1 and T6SS-2 (Figure 1). T6SS-1 appears to be complete as it contains *hcp* (named *hcp1*) secreted protein-encoding gene as well as the *clpV* ATPase-encoding gene (named *clpV1*), among other core and accessory gene components (Figure 1; Supplementary Table S1), suggesting that T6SS-1 could be functional in *E. cloacae*. In the T6SS-2 loci, the *hcp* core gene is missing; however, it contains genes such as *clpV* (named *clpV2*), *vgrG*, *PAAR*, *tssM*, and other core components (Figure 1; Supplementary Table S1). In both clusters, proteins homologous to T6SS components were found in enterobacteria such as *Salmonella enterica* serovar Typhimurium and enteroaggregative *Escherichia coli* (Supplementary Table S1).

**FIGURE 2:**
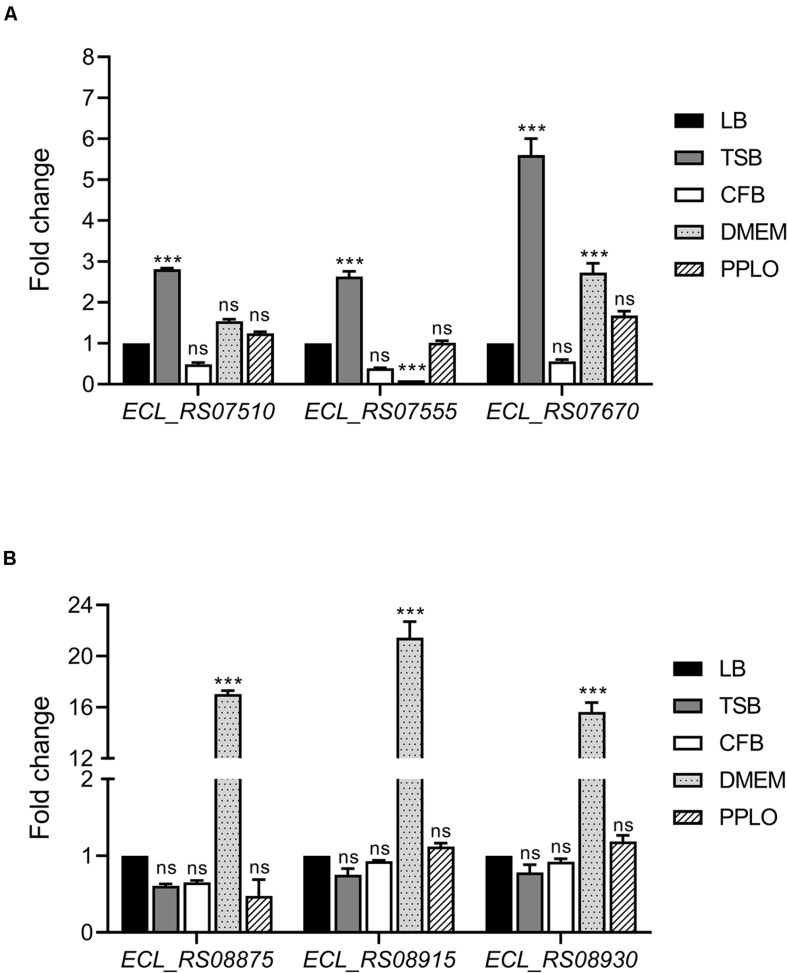
*E. cloacae* T6SS-1 and T6SS-2 are differentially expressed in TSB and DMEM. Fold change expression (RT-qPCR) of T6SS-1 **(A)** and T6SS-2 genes **(B)**, compared with LB medium. *E. cloacae* strains were grown in different culture media: lysogenic broth (LB), trypticase soy broth (TSB), Dulbecco’s Modified Eagle’s Medium (DMEM) with high glucose (4.5 g/l), pleuropneumoniae-like organisms (PPLO) broth, and colonization factor antigen (CFA) broth. Bacterial cultures were grown at 37°C for 6 h. 16S rRNA was used as a reference gene for normalization. Data represent the mean of three independent experiments performed in triplicate. Statistically significant with respect to the WT bacteria grown in LB medium; ns: not significant; ***p < 0.001.

We evaluated gene expression of both T6SS-1 and T6SS-2 on 6 h of growth of *E. cloacae* in different culture media by RT-qPCR, determining the mRNA levels of three different genes of each genetic cluster, which are the first genes of putative operons. Transcription of *ECL_RS07510*, *ECL_RS07555*, and *ECL_RS07670* genes, which encompass T6SS-1, was enhanced when *E. cloacae* was grown in TSB (Figure 2A). In contrast, *ECL_RS08875*, *ECL_RS08915*, and *ECL_RS08930* genes, belonging to T6SS-2, were highly expressed in DMEM (Figure 2B). Differences observed in genetic organization and expression in both gene clusters could suggest different roles of these secretion systems in the pathogenesis of *E. cloacae* ATCC 13047.

### T6SS-1 Is Required for Bacterial Competition

**FIGURE 3:**
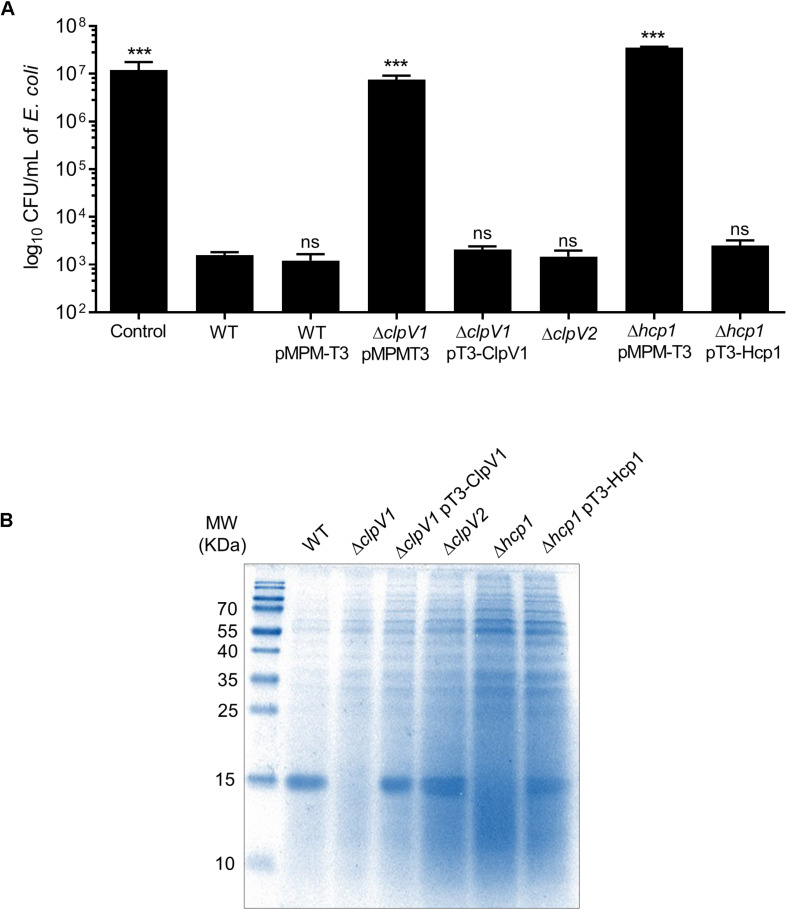
*E. cloacae* T6SS-1 is required for the bacterial competition. **(A)** Comparison of the survival of *E. coli* DH5α against wild-type *E. cloacae* and Δ*clpV1*, Δ*clpV2*, and Δ*hcp1* mutants. Survival rates are expressed in CFU/ml. Control: *E. coli* DH5α in LB with no *E. cloacae* strains. **(B)** Hcp1 protein was detected by 16% SDS-PAGE and Coomasie Blue staining, from concentrated supernatants of *E. cloacae* strains grown at 6 h in TSB medium. Statistically significant with respect to the wild-type strain; ns: not significant; ***p < 0.001.

Several findings reveal that T6SSs have bactericidal activity against different bacterial species (MacIntyre et al., 2010; Murdoch et al., 2011; Repizo et al., 2015). To investigate the role of both T6SSs in this phenotype, we initially focused in the deletion of *clpV1* and *clpV2* genes, which code for ATPases of each T6SS, and they are required for a functional system. *E. coli* DH5α was used as a target strain in the antibacterial competition assay. While wild-type *E. cloacae* strain was able to kill *E. coli*, the ability of Δ*clpV1* mutant to outcompete against *E. coli* was abolished (Figure 3A). In contrast, the absence of a functional T6SS-2, observed in the Δ*clpV2* mutant, did not affect the *E. cloacae* competition against *E. coli* (Figure 3A). As further confirmation of the contribution of the T6SS-1 to the killing of *E. coli*, the phenotype of the Δ*hcp1* mutant was also analyzed. The absence of the Hcp1 protein impaired the ability of *E. cloacae* to kill *E. coli*, showing a similar phenotype to the absence of ClpV1 ATPase (Figure 3A). Complementation of the Δ*hcp1* mutant restored *E. coli* killing to wild-type *E. cloacae* levels.

To demonstrate if T6SS-1 was functional, we performed protein secretion assays to detect the Hcp1 protein from supernatants recovered of bacterial culture grown at 6 h in TSB. A clear band was observed in the wild-type strain that it was absent in the Δ*clpV1* and the Δ*hcp1* mutants (Figure 3B). We detected the band in the complemented Δ*clpV1* and Δ*hcp1* mutants and the Δ*clpV2* single mutant. These 17-kDa bands were excised from Coomassie-stained gel and verified by LC-MS/MS analysis, corroborating the identity of the Hcp protein (Supplementary Table S2). These results demonstrate that killing of *E. coli* by *E. cloacae* and Hcp1 protein secretion are dependent on a functional T6SS-1.

### *E. cloacae* T6SS-1 Is Involved in the Bacterial Competition Against Different *E. coli* Pathotypes and Other Gram-Negative Pathogens

**FIGURE 4:**
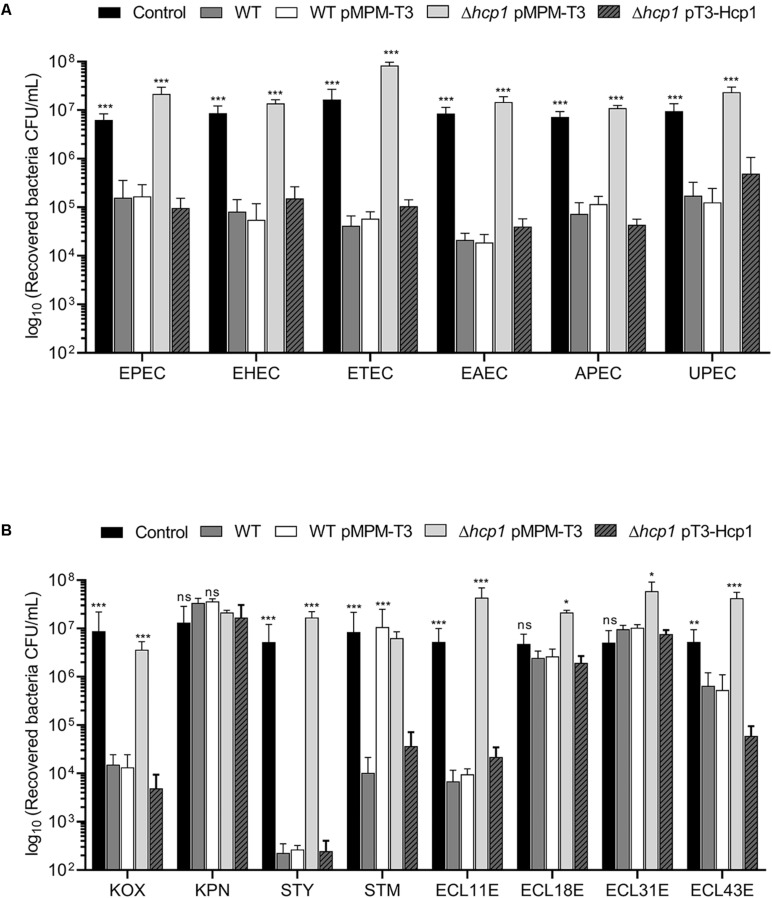
*E. cloacae* bacterial competition against different enteric bacteria. Comparison of the survival of the different *E. coli* pathotypes **(A)** and other Gram-negative pathogens **(B)** against wild-type *E. cloacae* (−/+pMPMT-3), Δ*hcp1* pMPM-T3, and Δ*hcp1* pT3-Hcp1. EPEC: enteropathogenic *E. coli*. EHEC: enterohemorragic *E. coli*. ETEC: enterotoxigenic *E. coli*. EAEC: enteroaggregative *E. coli*. APEC: avian-pathogenic *E. coli*. UPEC: uropathogenic *E. coli.* KOX: *K. oxytoca*. KPN: *K. pneumoniae.* STY: *S.* Typhi. STM: *S.* Typhimurium. ECL11E, ECL18E, ECL31E, and ECL43E: clinical isolates of *E. cloacae*. Survival levels are expressed in CFU/ml. Control: prey bacteria in LB with no *E. cloacae* strains. Statistically significant with respect to the wild-type strain; ns: not significant; *p < 0.05; **p < 0.01; ***p < 0.001.

We wanted to explore the impact of the antibacterial activity of *E. cloacae* on diarrheagenic and non-diarrheagenic pathotypes of *E. coli*, such as enteropathogenic (EPEC), enterohemorrhagic (EHEC), enterotoxigenic (ETEC), enteroaggregative (EAEC), uropathogenic (UPEC), and avian-pathogenic (APEC) *E. coli*. As shown in Figure 4A, wild-type *E. cloacae* strain caused a reduction between 10^2^- and 10^3^-fold in killing of the EPEC, EHEC, ETEC, EAEC, APEC, and UPEC strains. Interestingly, this loss of viability of *E. coli* pathotypes was caused by the *E. cloacae* T6SS-1 because the absence of Hcp1 resulted in the lack of killing activity of *E. cloacae* (Figure 4A). The complemented Δ*hcp1* mutant restored the antibacterial activity of *E. cloacae* against *E. coli* pathotypes.

In addition to *E. coli*, we analyzed the ability of *E. cloacae* to kill different Gram-negative enteropathogens such as *Salmonella*, *Klebsiella*, and other *E. cloacae* strains isolated from blood culture (Figure 4B). We found that wild-type *E. cloacae* was able to decrease the growth of *S.* Typhi (∼10^4^-fold). For *S.* Typhimurium and *K. oxytoca*, *E. cloacae* decreased the bacterial viability by 10^3^-fold. Interestingly, *E. cloacae* ATCC 13047 killed two *E. cloacae* clinical isolates around 10^1^- and 10^3^-fold. Surprisingly, *K. pneumoniae* and two *E. cloacae* clinical isolates did not show a reduction in the recovery of viable cells in presence of wild-type *E. cloacae* (Figure 4B). Our data show that T6SS-1 confers an advantage to *E. cloacae* in an inter- and intra-bacterial competition against other bacterial pathogens.

### T6SS-1 Confers Hemolytic Activity to *E. cloacae*

**FIGURE 5:**
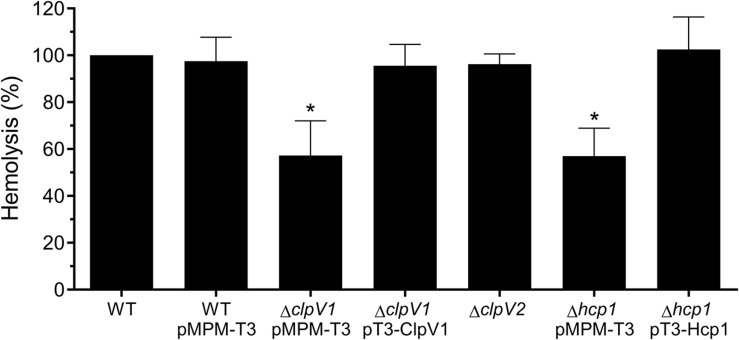
Hemolysis assay describing T6SS-1-mediated hemolytic activity of *E. cloacae*. Hemolysis of wild-type *E. cloacae* (−/+pMPM-T3), Δ*clpV1* pMPM-T3, Δ*clpV1* pT3-ClpV1, Δ*clpV2*, Δ*hcp1* pMPM-T3, and Δ*hcp1* pT3-Hcp backgrounds was quantified by measuring absorbance at 450 nm and expressed in percentage with respect to the lysis obtained with wild-type *E. cloacae* strain. Statistically significant with respect to the wild-type strain; ns: not significant; *p < 0.05.

Since T6SS confers hemolytic activity in some bacteria (Bleumink-Pluym et al., 2013; Zong et al., 2019), we analyzed this phenotype incubating red blood cells with wild-type *E. cloacae*, Δ*clpV1* pMPM-T3, Δ*clpV1* pT3-ClpV1, Δ*clpV2* Δ*hcp1* pMPM-T3, and Δ*hcp1* pT3-Hcp1 mutants. The lack of a functional T6SS-1 impaired the hemolytic activity of *E. cloacae* (Figure 5). Likewise, the absence of Hcp1 protein diminished to 43% the bacterial hemolytic activity compared with the wild-type *E. cloacae* (Figure 5). The complemented Δ*clpV1* and Δ*hcp* mutants restored the hemolysis levels. These results demonstrate that Hcp1 protein secretion, which is T6SS-1 dependent, confers hemolytic activity to *E. cloacae*.

### T6SS-2 Is Implicated in Biofilm Formation and Adherence to Epithelial Cells

**FIGURE 6:**
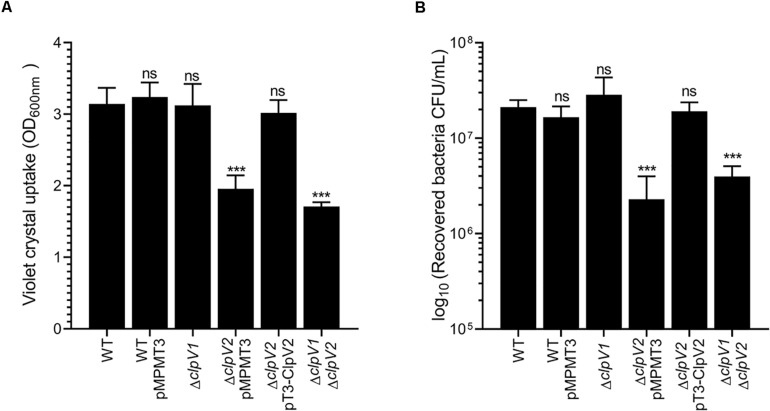
Role of *E. cloacae* T6SSs on the biofilm formation and cell adherence. **(A)** Quantification of biofilm formation by measuring crystal violet uptake. Wild-type *E. cloacae* and T6SS mutants were grown for 24 h in DMEM. **(B)** Adherence of wild-type *E. cloacae* Δ*clpV1*, Δ*clpV2*, Δ*clpV2* pT3-ClpV2, and Δ*clpV1* Δ*clpV2* backgrounds, after 2 h of infection of HeLa cell monolayers. Statistically significant differences between wild-type *E. cloacae* and their respective T6SS isogenic mutants; ns: not significant; ***p < 0.001.

A virulence hallmark of Proteobacteria is their ability to interact to both abiotic and biotic surfaces, resulting mainly in the phenotypes of biofilm formation and adherence to epithelial cells, respectively, which are crucial for pathogenicity. In the case of *E. cloacae*, it was suggested that adherence to epithelial cells and biofilm formation are traits associated with pathogenicity (Krzyminska et al., 2010; Mezzatesta et al., 2012). To explore the roles of both *E. cloacae* T6SSs in these phenotypes, we evaluated both cell adherence and biofilm formation of the wild-type *E. cloacae* and T6SS-ATPases mutant strains (Δ*clpV1* and Δ*clpV2*). We observed that only Δ*clpV2* mutant strain exhibited a significant decrease in the biofilm formation compared with wild-type strain (Figure 6A). The Δ*clpV2* complemented mutant, which expresses *clpV2* gene from a *lac* promoter, was able to restore biofilm formation to levels similar to the wild-type strain (Figure 4A). Furthermore, the adherence of Δ*clpV2* single mutant to HeLa cells was also reduced (11.3-fold) compared to wild-type strain (Figure 6B). While a Δ*clpV1* Δ*clpV2* double mutant showed the same phenotype that a Δ*clpV2* single mutant, the Δ*clpV1* mutant was similar to the wild-type strain indicating that T6SS-1 was neither required for adherence to HeLa cells nor for biofilm formation (Figure 6). We evaluated the probable role of T6SS on macrophage-mediated phagocytosis. Neither T6SS-1 nor T6SS-2 participated in resistance to phagocytosis (Supplementary Figure S2). The data compellingly show that the *E. cloacae* T6SS-2 contributes to adherence to both abiotic and biotic surfaces.

### Both T6SSs of *E. cloacae* Contribute for the Bacterial Colonization *in vivo*

**FIGURE 7:**
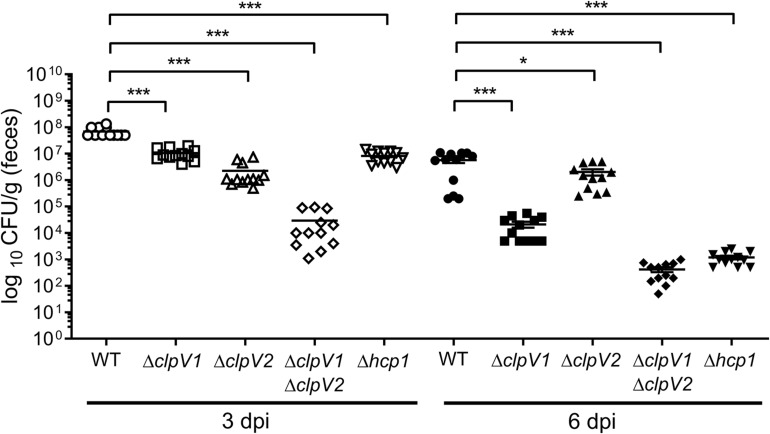
Both T6SSs of *E. cloacae* are required for the gut colonization. BALB/c mice were infected by intragastric inoculation with 10^9^ CFU/ml with the WT strain and their respective isogenic mutants in the ATPases of both T6SSs. Bacterial colonization was assessed after 3 and 6 days post-infection. Statistically significant differences between wild-type *E. cloacae* and their respective T6SS isogenic mutants; *p < 0.05; ***p < 0.001.

Given that the T6SS is associated with bacterial pathogenesis, we investigated the *in vivo* contribution of both *E. cloacae* T6SSs in colonization of the mouse gut. For this purpose, BALB/c mice were infected with wild-type *E. cloacae* strain and the Δ*clpV1*, Δ*clpV2* and Δ*clpV1* Δ*clpV2* mutants and bacteria were recovered 3 and 6 days post-infection (Figure 7). After 3 days post-infection, the Δ*clpV1* and Δ*clpV2* strains showed a decrease in colonization by 74.62- and 9.68-fold, respectively, compared with the wild-type strain. The Δ*hcp1* mutant resulted in a similar phenotype than the Δ*clpV1* single mutant. However, very low CFU numbers were recovered in the Δ*clpV1* Δ*clpV2* double mutant compared with Δ*clpV1*, Δ*clpV2*, and Δ*hcp1* single mutants (Figure 7).

At day 6 post-infection, the absence of ClpV1 and Hcp1 also reduced colonization levels (272.38-fold) of *E. clocace* (Figure 7). The CFU numbers of Δ*clpV2* single mutant had a slight decrease (2.82-fold) compared with the wild-type strain. Interestingly, the Δ*clpV1* Δ*clpV2* double mutant was strongly affected in the colonization of mice gut compared with Δ*clpV1* single mutant (Figure 7). These data strongly indicate that both T6SSs are required for intestinal colonization.

## Discussion

The type VI secretion system was initially described in *Vibrio cholerae* (Pukatzki et al., 2006); however, these genes have been found in more than 25% of all sequenced Gram-negative bacteria (Boyer et al., 2009). Recently, it was reported that *E. cloacae* strains possess gene sequences that code for at least one T6SS (Liu et al., 2013). This work represents the first investigation of the T6SS function in the *Enterobacter* genus and it describes the different roles of two T6SS in the pathogenicity of *E. cloacae* ATCC 13047. Several studies have demonstrated that environmental conditions are important for the differential expression of genes involved in the bacterial virulence (Dong and Mekalanos, 2012; Blair et al., 2013; De La Cruz et al., 2017; Ares et al., 2019). We showed that TSB and DMEM growth media stimulated the expression of *E. cloacae* T6SS-1 and T6SS-2, respectively, indicating that gene expression in both T6SSs is regulated by specific environmental cues, most likely compounds present in the culture medium. Interestingly, the expression of *ECL_RS07670* was slightly increased in DMEM. *ECL_RS07670-ECL_RS7700* genes form a putative polycistronic operon, and *ECL_RS07695* codes for an Rhs toxin, which could be involved in bacteria–bacteria and bacteria–eukaryotic cell interactions, as it was reported for *Dickeya dadantii* and *S*. Typhimurium, respectively (Koskiniemi et al., 2013; Starsta et al., 2020). Hence, DMEM components might mimic conditions that occur during the bacteria–cell host interaction and it would stimulate the transcription of *ECL_RS07670-ECL_RS7700* operon in such conditions.

T6SS core structural constituents play an important role in bacteria–bacteria interactions (Wexler et al., 2016). One crucial component of the T6SS is the Hcp protein, which constitutes the ∼600-nm-long inner tube wrapped into a sheath-like structure and is essential for the translocation of effector proteins (Basler et al., 2012; Brunet et al., 2014; Zoued et al., 2014). When the tail sheath is contracted, the components are recycled by the ClpV ATPase for a new cycle of T6SS tail elongation (Douzi et al., 2016). The absence of Hcp1 or ClpV1 decreased the ability of *E. cloacae* to outcompete against pathogenic and non-pathogenic *E. coli* and other Gram-negative bacteria as well (including other *E. cloacae* clinical isolates), supporting the notion that T6SS-1 functions as an antibacterial weapon that could be used by *E. cloacae* to compete against other bacteria. This inter- and intra-bacterial competition that is T6SS dependent has been described for *Acinetobacter baumannii*, another Gram-negative opportunistic pathogen (Repizo et al., 2015). Interestingly, *E. cloacae* was not able to kill *K. pneumoniae* or the two *E. cloacae* clinical isolates. Ongoing work by our group shows that the capsule polysaccharide protects *K. pneumoniae* against the T6SS of other bacteria, acting as a shield (Soria-Bustos et al., in preparation; Bleumink-Pluym et al., 2013; Toska et al., 2018). One explanation for the resistance of the two *E. cloacae* clinical isolates is that these bacteria possess homologous antitoxin proteins encoded in the T6SS clusters, which neutralize the poison proteins of *E. cloacae* ATCC 13047.

An interesting structural feature of the T6SS-2 is that it lacks the canonical Hcp protein. Besides, Hcp1 protein was not required for the T6SS-2 activity because the cell adherence and biofilm formation in Δ*hcp1* were similar to wild-type strain (Supplementary Figure S3). However, four Hcp1 homologous proteins were found in the genome of *E. cloacae* ATCC 13047: Hcp2 (ECL_RS00165), Hcp3 (ECL_RS07685), Hcp4 (ECL_RS10590), and Hcp5 (ECL_RS19875), which showed 26 and 45% amino acid identity and similarity, respectively. The role of those Hcp1 homologs in the function of *E. cloacae* T6SSs remains unknown. It is reasonable to speculate that any of these proteins could be associated with the T6SS-2 needle structure. Therefore, the mutations of such *hcp1* homologous genes and the resulting phenotypic effects are currently researched in our group.

Our results show that the *E. cloacae* T6SS-2 plays an essential role in biofilm formation and adherence to eukaryotic cells. These observations are in agreement to the function of T6SS of other pathogens on the bacterial adherence on abiotic and biotic surfaces (de Pace et al., 2011; Lertpiriyapong et al., 2012; Sheng et al., 2013; Tang et al., 2015; Gallique et al., 2017). Enteroaggregative *E. coli* strain 17-2 codes for two functional T6SS named Sci-1 and Sci-2, which are both involved in bacterial competition (Brunet et al., 2013; Flaugnatti et al., 2016), but Sci-1 also confers the ability to form biofilms (Aschtgen et al., 2008). Recently, it was reported that the absence of two functional T6SSs in *K. pneumoniae* affected the expression of type-1 fimbriae and subsequently the adherence to epithelial cells was impaired (Hsieh et al., 2019). Ongoing experiments in our laboratory will analyze if T6SS-2 controls the expression of type-1 fimbriae or any other adherence factor in *E. cloacae*. In terms of macrophage interaction, T6SS confers phagocytosis resistance in some bacteria (Suarez et al., 2008; Wan et al., 2017). Our data showed that neither T6SS-1 nor T6SS-2 are involved in this phenotype.

The T6SS has been shown to be relevant for virulence *in vivo* in many pathogenic bacteria (Lertpiriyapong et al., 2012; Repizo et al., 2015; Sana et al., 2016; Hsieh et al., 2019; Ungureanu et al., 2019). Here, we demonstrated the importance of both *E. cloacae* T6SSs in intestinal colonization of BALB/c mice. Both Δ*clpV1* and Δ*clpV2* mutants showed reduced levels of colonization as demonstrated by a reduced number of colony-forming units recovered in feces as compared with the wild-type strain. However, the absence of a functional T6SS-1 (e.g., in the Δ*hcp1* or Δ*clpV1* mutants) had a higher effect in colonization than the absence of the T6SS-2. Interestingly, both *E. cloacae* T6SSs showed an additive effect in *in vivo* intestinal colonization, suggesting that both secretion systems are important in the adherence of *E. cloacae* to epithelial cells and, most importantly, in the bacterial competition of this microorganism against other bacteria found in the gut microbiota.

Phylogenetically, *E. cloacae* T6SS-1 is related to the *S.* Typhi and *S*. Typhimurium T6SSs located on *Salmonella* Pathogenicity Island 6, which are required for gut colonization and systemic infection in humanized and non-humanized mice, respectively (Libby et al., 2010; Pezoa et al., 2013; Sana et al., 2016). In contrast, T6SS-2 genes such as *clpV*, *vgrG*, *PAAR*, and *tssM*, were phylogenetically associated with bacteria found in plants and soil (Liu et al., 2013). The outcome of this study supports a functional role of T6SS-1 and T6SS-2 in intestinal colonization and interactions with abiotic/biotic surfaces, respectively, which is likely to increase the ecological fitness of this microorganism.

In summary, our results demonstrate that the T6SS mutants were hampered in host cell adhesion and biofilm formation, antibacterial activity against multiple pathogens, and importantly, in intestinal colonization in mice. This study uncovers for the first time the presence and function of two T6SSs in *E. cloacae*, a trait that increases adaptability of this organism to different niches and hosts as part of their pathogenesis scheme.

## Data Availability Statement

All datasets presented in this study are included in the article/Supplementary Material.

## Ethics Statement

The animal study was reviewed and approved by the Internal Ethics Committee of the Animal Resource Facility of the Universidad Autónoma del Estado de Hidalgo (Approval number: CICUAL/016/2019R to CG-A).

## Author Contributions

JS-B and MD conceived and designed the experiments. JS-B and MA performed the experiments. JS-B, MA, CG-A, JG-M, JG, and MD analyzed the data. JS-B, JG, and MD wrote the manuscript. All authors contributed to the article and approved the submitted version.

## Conflict of Interest

The authors declare that the research was conducted in the absence of any commercial or financial relationships that could be construed as a potential conflict of interest.

## Footnotes

1 http://www.bioinformatics.nl/cgi-bin/primer3plus/primer3plus.cgi/

2 https://www.ncbi.nlm.nih.gov/refseq/about/non-redundantproteins/

3 http://prospector.ucsf.edu/prospector

## Supplementary Material

FIGURE S1 Expression of reference gene (*rrsH*) under different bacteriological broths. Quantification of expression is showed as copies of *rrsH*/μg RNA.

FIGURE S2 Phagocytosis resistance of wild-type *E. cloacae*, Δ*clpV1*, Δ*clpV2*, and Δ*hcp1* mutants. Comparison of phagocytosis levels between the different strains using THP-1 human monocyte-derived macrophages. Statistically significant differences between wild-type *E. cloacae* and their respective T6SS isogenic mutants; ^∗∗∗^*p* < 0.001.

FIGURE S3 Hcp1 does not affect the biofilm formation and cell adherence. Comparison of biofilm formation and cell adherence levels between the Δ*hcp1* mutant and the wild-type strain.
