# Supplementary figures and images for: Two Type VI Secretion Systems of Enterobacter cloacae Are Required for Bacterial Competition, Cell Adherence, and Intestinal Colonization
Source: Front Microbiol. 2020 Sep 24;11:560488. doi: 10.3389/fmicb.2020.560488 (PMC7541819; doi:10.3389/fmicb.2020.560488)

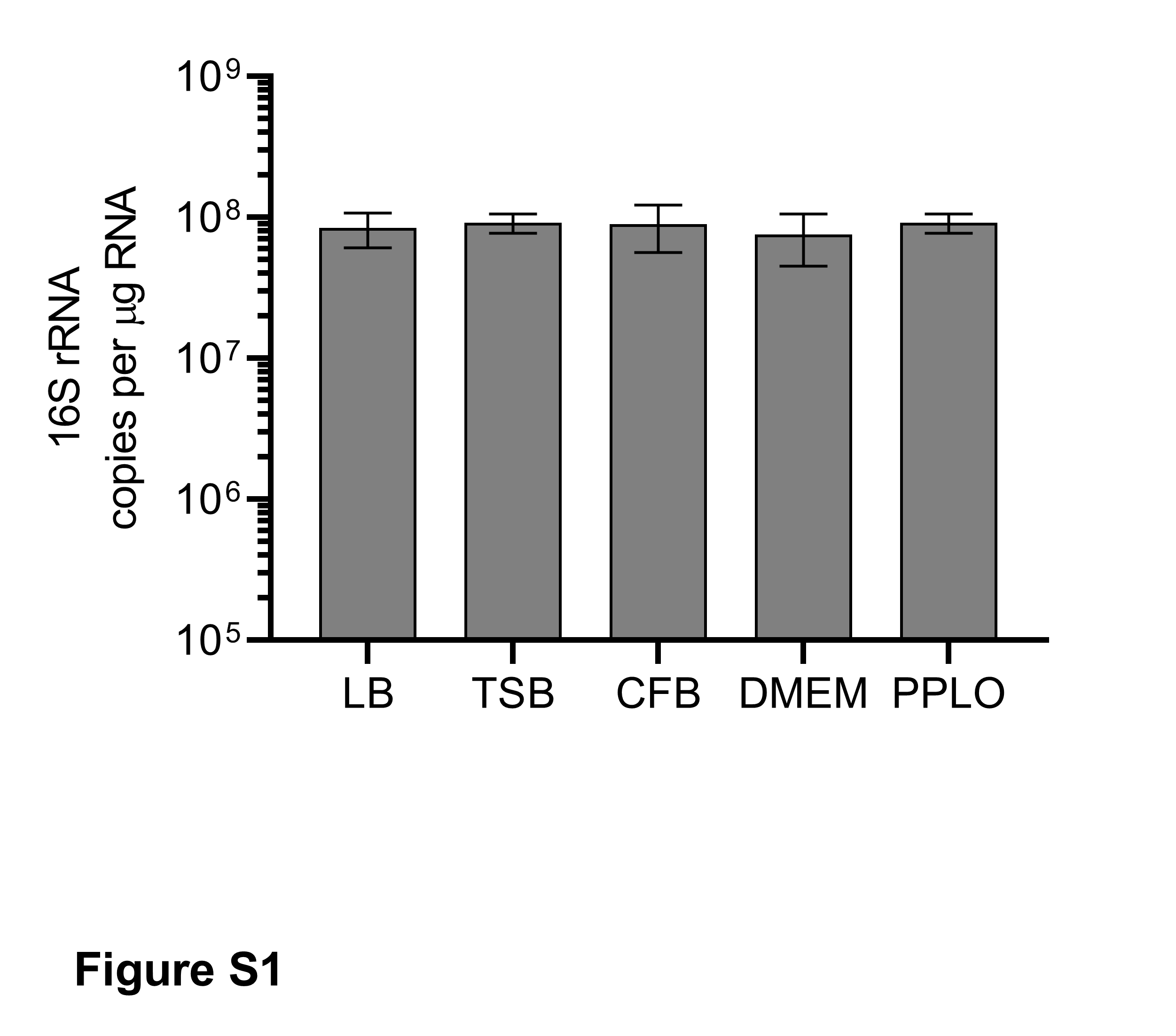

Supplement: FIGURE S1 — Expression of reference gene (rrsH) under different bacteriological broths. Quantification of expression is showed as copies of rrsH/μg RNA. [file Image_1.TIF]

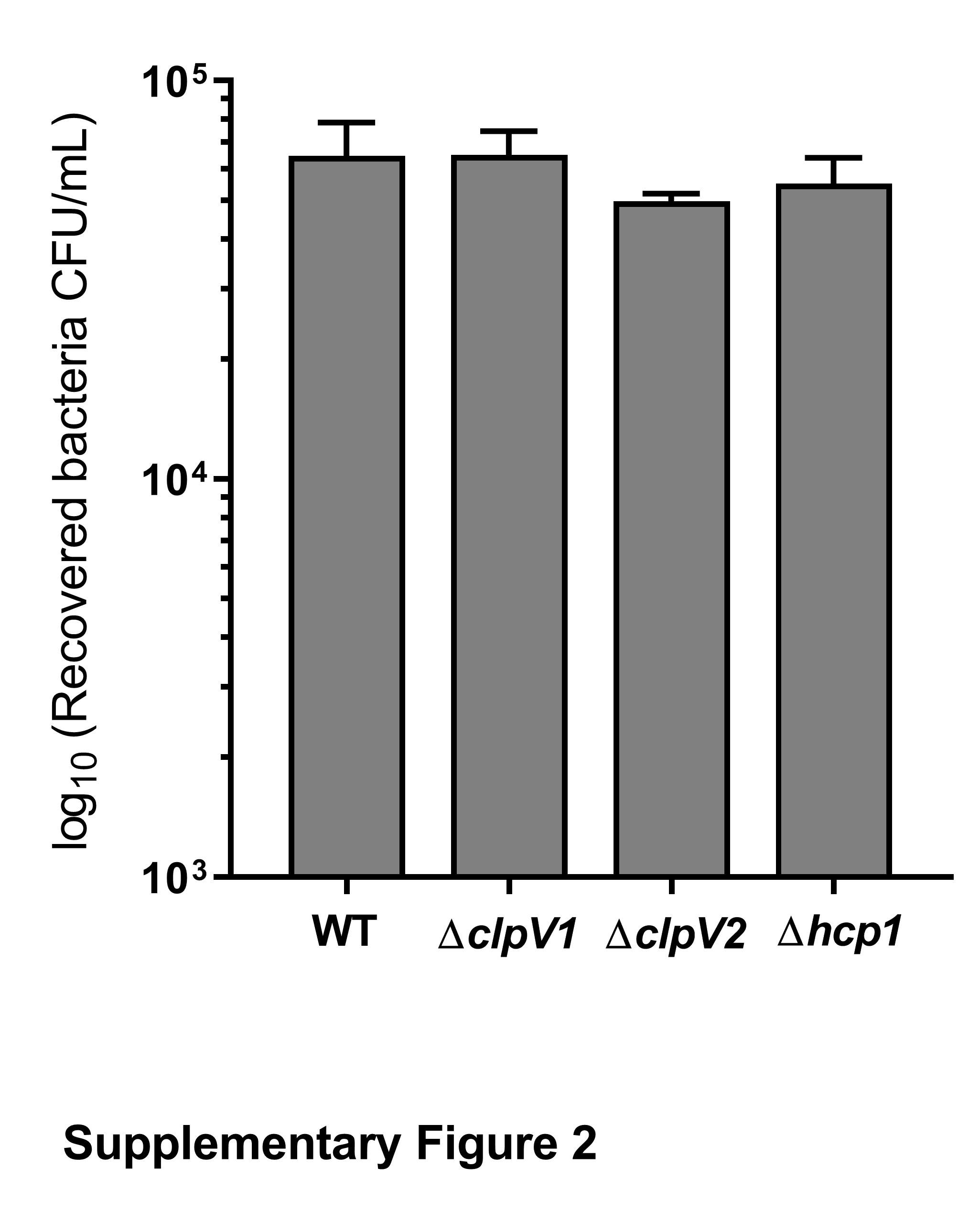

Supplement: FIGURE S2 — Phagocytosis resistance of wild-type E. cloacae, ΔclpV1, ΔclpV2, and Δhcp1 mutants. Comparison of phagocytosis levels between the different strains using THP-1 human monocyte-derived macrophages. Statistically significant differences between wild-type E. cloacae and their respective T6SS isogenic mutants; ∗∗∗p < 0.001. [file Image_2.TIF]

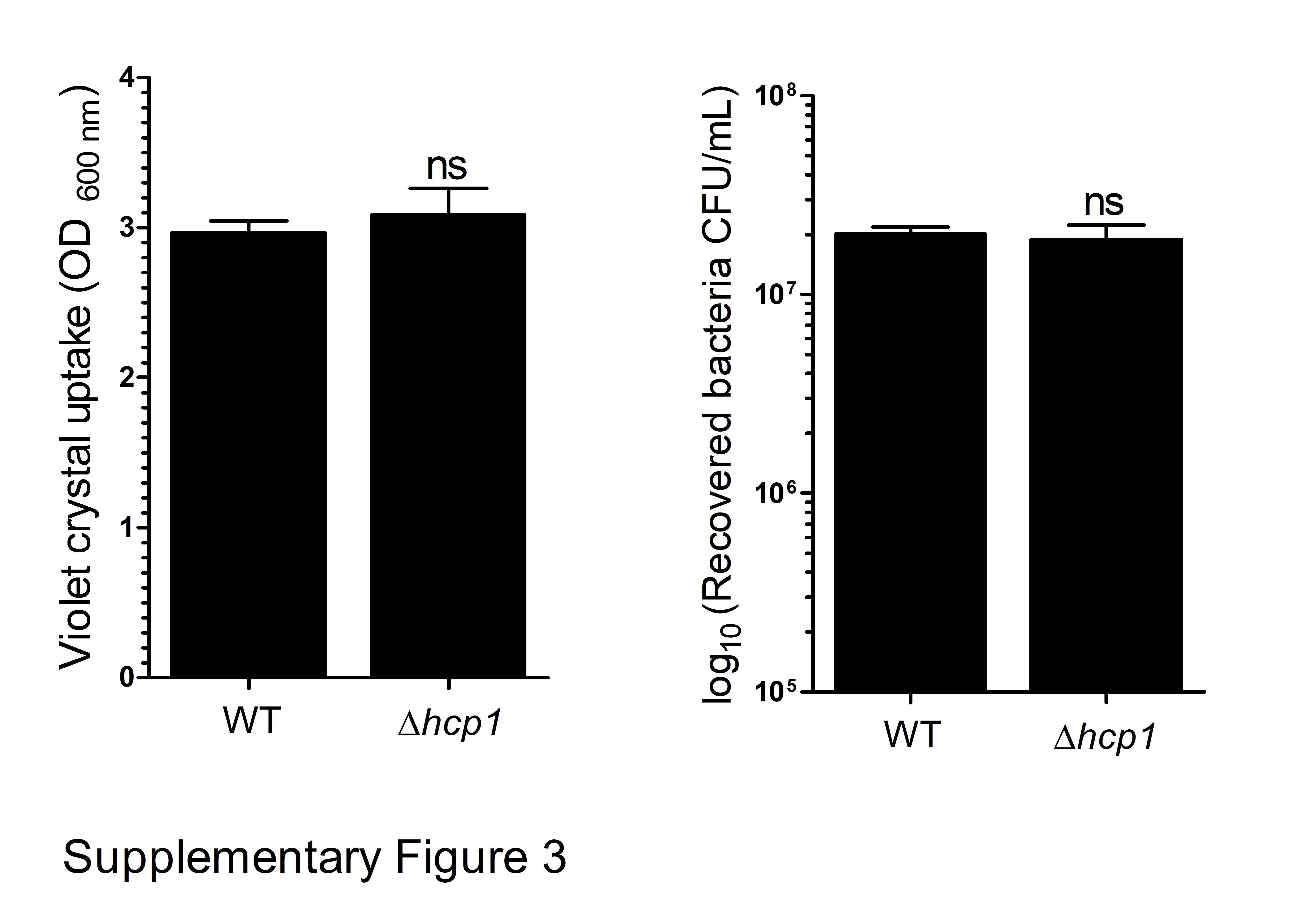

Supplement: FIGURE S3 — Hcp1 does not affect the biofilm formation and cell adherence. Comparison of biofilm formation and cell adherence levels between the Δhcp1 mutant and the wild-type strain. [file Image_3.TIF]
