# Supplementary material for: Two Type VI Secretion Systems of Enterobacter cloacae Are Required for Bacterial Competition, Cell Adherence, and Intestinal Colonization
Source: Front Microbiol. 2020 Sep 24;11:560488. doi: 10.3389/fmicb.2020.560488 (PMC7541819; doi:10.3389/fmicb.2020.560488)
Supplement: Supplementary file 4 [file Data_Sheet_1.PDF]

Table S1. Homology between *E. cloacae* T6SS proteins and other bacteria

T6SS-1

Gene Ecl	Name	Function	Homologous	Bacteria	locus	% Identity	% Similarity
**ECL_RS07515**	TssJ (VasD)	Membrane complex lipoprotein	Hypothetical protein STM14_1514	*S.* Typhimurium 14028S	ACY87994.1	41	77
**ECL_RS07520**	TssK	Baseplate subunit	Putative T6SS protein	*E. coli* str. 042	CBG33049.1	33	52
			Putative cytoplasmic protein	*S.* Typhimurium 14028S	ACY86855.1	33	52
**ECL_RS07525**	TssL	DotU family T6SS	Hhypothetical protein STM14_0330	*S.* Typhimurium 14028S	ACY86856.1	38	53
			Putative T6SS protein	*E. coli* str. 042	CBG33048.1	35	49
**ECL_RS07530**	TssM ImcF impL	Membrane complex subunit	Putative inner membrane protein	*S.* Typhimurium 14028S	ACY86859.1	32	48
			Putative T6SS protein	*E. coli* str. 042	CBG33044.1	26	41
**ECL_RS07540**	TssA impA	Cap	Putative cytoplasmic protein	*S.* Typhimurium 14028S	ACY86839.1	25	42
			putative T6SS protein	*E. coli* str. 042	CBG37392.1	25	42
			Putative T6SS protein	*E. coli* str. 042	CBG37362.1	38	52
**ECL_RS07545**	TssB	Contractile sheath small subunit	Putative cytoplasmic protein	*S.* Typhimurium 14028S	ACY86846.1	73	86

			Putative T6SS protein	*E. coli* str. 042	CBG37383.1	43	57
**ECL_RS07550**	TssC	Sheath large subunit	Putative cytoplasmic protein	*S.* Typhimurium 14028S	ACY86847.1	71	84
			Putative T6SS protein	*E. coli* str. 042	CBG37384.1	45	65
**ECL_RS07555**	Hcp	Inner tube	Putative cytoplasmic protein	*S.* Typhimurium 14028S	ACY86850.1	38	57
**ECL_RS07560**	Tae4	Effector amidase	Putative cytoplasmic protein	*S.* Typhimurium 14028S	ACY86851.1	54	68
**ECL_RS07565**	Tai4	Antitoxin	Putative periplasmic protein	*S.* Typhimurium 14028S	ACY86852.1	26	49
**ECL_RS07585**	TagJ		Putative cytoplasmic protein	*S.* Typhimurium 14028S	ACY86843.1	33	49
**ECL_RS07590**	TssE	Baseplate	Putative cytoplasmic protein	*S.* Typhimurium 14028S	ACY86842.1	38	58
**ECL_RS07595**	TssF	Baseplate	Putative cytoplasmic protein	*S.* Typhimurium 14028S	ACY86841.1	44	60
			Putative T6SS protein	*E. coli* str. 042	CBG37387.1	27	45
**ECL_RS07600**	TssG	Baseplate	Putative cytoplasmic protein	*S. enterica* serotype Typhimurium 14028S	ACY86840.1	39	56
			TssG	*E. coli* str. 042	CBG37388.1	25	40
**ECL_RS07605**	TssH ClpV1	ATPase	Putative chaperone ATPase	*S.* Typhimurium 14028S	ACY86845.1	55	69
			Putative T6SS protein	*E. coli* str. 042	CBG37352.1	46	63

**ECL_RS07625**		Hypothetical protein	Hypothetical protein ECL_01556	*E. cloacae* ATCC 13047	ADF61115.1	36	57
			Putative membrane protein	*E. coli* str. 042	CBG34215.1	53	63
**ECL_RS07630**		IS5-like element IS903B family transposase	Hypothetical protein FVF73_22345	*E. coli* str. 042	QEG53185.1	64	82
**ECL_RS07645**	TssI VgrG	Tip protein	Putative cytoplasmic protein	*S.* Typhimurium 14028S	ACY86864.1	36	51
			T6SS Rhs/Vgr-family protein	*E. coli* str. 042	CBG33059.1	33	51
**ECL_RS07650**		ADP-ribosylglycohydrolase family protein	ADP-ribosylglycohydrolase family protein	*E. coli* str. 042	QEG51093.1	53	13
			Putative glycohydrolase	*S.* Typhimurium 14028S	ACY89087.1	41	53
			Hypothetical protein FVF73_22345	*E. coli* str. 042	QEG53185.1	64	82
**ECL_RS07695**	Rhs	T6SS tip?	Putative RHS-like protein	*S.* Typhimurium 14028S	ACY86866.1	32	45
			RHS repeat protein	*E. coli* str. 042	QEG49802.1	26	39

T6SS-2							
**Gene Ecl**	**Name**	**Function**	**Homologous**	**Bacteria**	**locus**	**% Identity**	**% Similarity**
**ECL_RS08875**	TssB	Contractile sheath small subunit	Putative T6SS protein	*E. coli* str. 042	CBG37346.1	70	85
			Putative cytoplasmic protein	*S.* Typhimurium 14028S	ACY86846.1	31	54
**ECL_RS08880**	TssC	Contractile sheath large subunit	Putative T6SS protein	*E. coli* str. 042	CBG37347.1	82	91
			Putative cytoplasmic protein	*S.* Typhimurium 14028S	ACY86847.1	40	60
**ECL_RS08885**	TssK	T6SS baseplate subunit	Putative T6SS protein	*E. coli* str. 042	CBG37348.1	57	70
			Putative cytoplasmic protein	*S.* Typhimurium 14028S	ACY86855.1	24	40
**ECL_RS08890**	DotU family type IV/VI secretion system protein	Putative OmpA-family membrane protein	T6SS protein	*E. coli* str. 042	CBG37349.1	38	55
**ECL_RS08930**	hypothetical protein		Putative structural protein	*E. coli* str. 042	QEG53248.1	43	70
**ECL_RS08935**	TssM<br>ImpL	Membrane complex protein	Putative T6SS protein	*E. coli* str. 042	CBG37361.1	44	60
**ECL_RS08940**	TssA	Cap	Chain A, Tssa	*E. coli* str. 042	4YO3_A	69	81
			Putative T6SS protein	*E. coli* str. 042	CBG33045.1	25	40

**ECL_RS08960**	TssG	Baseplate subunit	Putative T6SS protein	*E. coli* str. 042	CBG37365.1	47	59
			Putative cytoplasmic protein	*S. enterica* serotype Typhimurium 14028S	ACY86840.1	32	46
**ECL_RS08965**	TssJ	Membrane complex lipoprotein	Putative T6SS protein	*E. coli* str. 042	CBG37366.1	48	65
			Putative outer membrane lipoprotein	*S.* Typhimurium 14028S	ACY86854.1	23	47
**ECL_RS08970**	TssE	Baseplate subunit	Putative T6SS protein	*E. coli* str. 042	CBG37367.1	74	85
**ECL_RS08975**	TssA<br>VasL	Cap	Putative T6SS protein	*E. coli* str. 042	CBG33043.1	30	46
