# Supplementary material for: Two Type VI Secretion Systems of Enterobacter cloacae Are Required for Bacterial Competition, Cell Adherence, and Intestinal Colonization
Source: Front Microbiol. 2020 Sep 24;11:560488. doi: 10.3389/fmicb.2020.560488 (PMC7541819; doi:10.3389/fmicb.2020.560488)
Supplement: Supplementary file 5 [file Data_Sheet_2.PDF]

Table S2. LC-MS/MS-generated peptides.

Protein band	GenBank Accession* Protein name	Peptides generated by LC-MS/MS	
**WT**	**ADF61100.1**	**Coverage 75.8%; 10 peptides**	**Discriminant Score[¥]**
	Type VI secretion system secreted protein Hcp [Enterobacter cloacae subsp. cloacae ATCC 13047]	TGESKDSNHTGWTDITSFSWGASQPG	31.1
		DLHVNALIDKSTTAILK	52.9
		LEDVLVTSVQYTGADNGDT	52.3
		VGVTYAFQAAKVKQQYW	44.5
		DNGDTVGVTYAFQAAKVKQQYW	30.6
		GQQVEYSRIT	39.2
		LEDVLVTSVQYTGA	39.8
		VELSVCKAG	30.8
		MSVGGGGG	38.3
		VGGGGGAGK	27.8
∆clpV1 **pT3-ClpV1**	**ADF61100.1**	**Coverage 32.7%; 5 peptides**	**Discriminant Score[¥]**
	Type VI secretion system secreted protein Hcp [Enterobacter cloacae subsp. cloacae ATCC 13047]	FLKVEGVTGESKDSNHTGWTDITSF	55.8
		GVTGESKDSNHTGWTDITSFSWGASQ	54.7
		SGKHLTKVELSVCKAGGQQVEYS	32.9
		VEYSRITLEDVLV	36.5
		AFQAAKVKQQYWE	22.9
∆clpV2	**ADF61100.1**	**Coverage 35.8%; 7 peptides**	**Discriminant Score[¥]**
	Type VI secretion system secreted protein Hcp [Enterobacter cloacae subsp. cloacae ATCC 13047]	VEGVTGESKDSNHTGWTDI	52.2
		VTGESKDSNHTGW	48.3
		VGGGGGAGKVNFNDLH	32.3
		QVEYSRITLED	49.8
		EDVLVTSVQYTGA	35.4
		KQQYWEQTTS	33.7
		GKGAESSAGWNI	38.8

∆hcp1 **pT3-Hcp1**	**ADF61100.1**<br><br>Type VI secretion system secreted protein Hcp [Enterobacter cloacae subsp. cloacae ATCC 13047]	**Coverage 78.7%; 12 peptides**<br><br>GESKDSNHTGWTDITSFSWGASQ<br>YTGADNGDTVGVTYAF<br>DITSFSWGASQPGNMSVGGGG<br>KHCASGKHLTKVELSV<br>NDLHVNALIDKSTTAILKHCAS<br>STTAILKHCASGKHLTKVEL<br>NGDTVGVTYAFQAAKVK<br>GGKGAESSAGWN<br>YWEQTTSGGKG<br>TGADNGDTVGVT<br>KGAESSAGWNI<br>GGKGAESSAG	**Discriminant Score[¥]**<br><br>59.8<br>58.3<br>50.8<br>41.8<br>51.9<br>53.8<br>56.0<br>41.0<br>47.5<br>40.4<br>38.5<br>16.2

*The GenBank Accession number is according to the NCBI protein database (https://www.ncbi.nlm.nih.gov/protein/)

[¥]The discriminant score is a number that is the combination of two measurements of the search result. One of them is the expectation value for the peptide match (a measure of the likelihood that a match is at random) and the other is a 'best peptide score', which takes into account the fact that if a protein has been confidently identified in a sample, it is more likely that other peptides will be identified from the same protein. As a default this threshold is set to 0 and, for a normal database search, discriminant scores below 0 are generally incorrect, whilst those above 0 are mostly correct. Protein Prospector™ version 5.10.17 (San Francisco CA, USA. http//prospector.ucsf.edu/prospector).
